# Supplementary material for: COMPASS: joint copy number and mutation phylogeny reconstruction from amplicon single-cell sequencing data
Source: Nat Commun. 2023 Aug 15;14:4921. doi: 10.1038/s41467-023-40378-8 (PMC10427627; doi:10.1038/s41467-023-40378-8)
Supplement: Supplementary file 3 — Reporting Summary [file 41467_2023_40378_MOESM3_ESM.pdf]

nature portfolio

Corresponding author(s): Katharina Jahn

Last updated by author(s): Jun 24, 2023

Reporting Summary

Nature Portfolio wishes to improve the reproducibility of the work that we publish. This form provides structure for consistency and transparency in reporting. For further information on Nature Portfolio policies, see our Editorial Policies and the Editorial Policy Checklist.

Statistics

For all statistical analyses, confirm that the following items are present in the figure legend, table legend, main text, or Methods section.

n/a	Confirmed	
☐	☒	The exact sample size (n) for each experimental group/condition, given as a discrete number and unit of measurement
☐	☒	A statement on whether measurements were taken from distinct samples or whether the same sample was measured repeatedly
☐	☒	The statistical test(s) used AND whether they are one- or two-sided<br>*Only common tests should be described solely by name; describe more complex techniques in the Methods section.*
☐	☒	A description of all covariates tested
☐	☒	A description of any assumptions or corrections, such as tests of normality and adjustment for multiple comparisons
☐	☒	A full description of the statistical parameters including central tendency (e.g. means) or other basic estimates (e.g. regression coefficient) AND variation (e.g. standard deviation) or associated estimates of uncertainty (e.g. confidence intervals)
☐	☒	For null hypothesis testing, the test statistic (e.g. F, t, r) with confidence intervals, effect sizes, degrees of freedom and P value noted<br>*Give P values as exact values whenever suitable.*
☐	☒	For Bayesian analysis, information on the choice of priors and Markov chain Monte Carlo settings
☒	☐	For hierarchical and complex designs, identification of the appropriate level for tests and full reporting of outcomes
☒	☐	Estimates of effect sizes (e.g. Cohen's d, Pearson's r), indicating how they were calculated

Our web collection on statistics for biologists contains articles on many of the points above.

Software and code

Policy information about availability of computer code

Data collection	No software was used for data collection.
Data analysis	The software developed in this study is available at https://github.com/cbg-ethz/COMPASS. The software is written in C++11, python was used for parts of the analysis, and snakemake pipelines were used to run the simulations. The python scripts and snakemake pipelines are also included in the GitHub repository. For the evaluation against other methods, we used the version of infSCITE available on GitHub on 1st June 2023 (https://github.com/cbg-ethz/infSCITE), and the version of BiTSC2 available on GitHub on 1st June 2023 (https://github.com/ucasdp/BITSC2). infSCITE and BiTSC2 do not have releases with version numbers. For the analysis of bulk SNP array data, we used ASCAT v3.1.0. For the analysis of bulk targeted sequencing, we used CNVkit v0.9.10.

For manuscripts utilizing custom algorithms or software that are central to the research but not yet described in published literature, software must be made available to editors and reviewers. We strongly encourage code deposition in a community repository (e.g. GitHub). See the Nature Portfolio guidelines for submitting code & software for further information.

Data

Policy information about availability of data

All manuscripts must include a data availability statement. This statement should provide the following information, where applicable:

- Accession codes, unique identifiers, or web links for publicly available datasets
- A description of any restrictions on data availability
- For clinical datasets or third party data, please ensure that the statement adheres to our policy

We did not generate any new experimental data, but used data from three published papers. All three datasets correspond to single-cell targeted DNA sequencing samples analyzed with MissionBio's Tapestri platform.
The first dataset consists of 123 AML samples profiled, from the publication from Morita et al. (Nature Communications, 2020). The raw sequencing data is available on the SRA with the project ID PRJNA648656. Our collaborators provided us with the loom files generated by the Tapestri pipeline.
The second dataset consists of 4 TP53-mutated AML samples, before and after venetoclax treatment, from the publication from Thijssen et al. (Blood, 2021). The data is not publicly available, but the authors shared the loom files with us.
The third datasets consists of 8 TP53-mutated MPN samples (Maslah et al., Blood advances 2022). The data is not publicly available, but the authors shared the loom files with us.
For all three datasets, the preprocessed data that is used as input for COMPASS was made available on GitHub (https://github.com/cbg-ethz/COMPASS/tree/master/data), with the agreement of the respective authors.

Human research participants

Policy information about studies involving human research participants and Sex and Gender in Research.

Reporting on sex and gender	The method that we developed is applicable to both male and female samples. The sex is given as input to the method, because it determines the number of sex chromosomes (XX or XY) in the absence of any somatic alteration.
Population characteristics	We used data that had already been generated. The main dataset that we used corresponds to adult patients diagnosed with acute myeloid leukemia.
Recruitment	We used data that had already been generated and did not recruit any patients. The main dataset that we used came from patients treated at the MD Anderson center.
Ethics oversight	Our work did not require approval of any organization. The main dataset that we used was generated at MD Anderson, and the ethics oversight was done by the University of Texas MD Anderson Cancer Center.

Note that full information on the approval of the study protocol must also be provided in the manuscript.

Field-specific reporting

Please select the one below that is the best fit for your research. If you are not sure, read the appropriate sections before making your selection.

☒ Life sciences ☐ Behavioural & social sciences ☐ Ecological, evolutionary & environmental sciences

For a reference copy of the document with all sections, see nature.com/documents/nr-reporting-summary-flat.pdf

Life sciences study design

All studies must disclose on these points even when the disclosure is negative.

Sample size	We did not perform sample size calculation and used all available data that we could find.
Data exclusions	We did not exclude any data.
Replication	We first developed our method while testing it on one dataset (Morita et al. 2020), and then validated that our method also worked on two additional datasets.
Randomization	No randomization was done. We did not assign individuals to different groups, therefore the randomization is irrelevant.
Blinding	There was no blinding.

Reporting for specific materials, systems and methods

We require information from authors about some types of materials, experimental systems and methods used in many studies. Here, indicate whether each material, system or method listed is relevant to your study. If you are not sure if a list item applies to your research, read the appropriate section before selecting a response.

Materials & experimental systems

n/a	Involved in the study
☒	☐ Antibodies
☒	☐ Eukaryotic cell lines
☒	☐ Palaeontology and archaeology
☒	☐ Animals and other organisms
☒	☐ Clinical data
☒	☐ Dual use research of concern

Methods

n/a	Involved in the study
☒	☐ ChIP-seq
☒	☐ Flow cytometry
☒	☐ MRI-based neuroimaging
